# Nutraceutical Strategies for Metabolic Dysfunction-Associated Steatotic Liver Disease (MASLD): A Path to Liver Health

**DOI:** 10.3390/nu17101657

**Published:** 2025-05-13

**Authors:** Emmanouil Vrentzos, George Pavlidis, Emmanouil Korakas, Aikaterini Kountouri, Loukia Pliouta, George D. Dimitriadis, Vaia Lambadiari

**Affiliations:** 1 4th Department of Internal Medicine, Medical School, Attikon University Hospital, National and Kapodistrian University of Athens, 12462 Athens, Greece; manos.vr@hotmail.com (E.V.); geo_pavlidis@yahoo.gr (G.P.); 2 2nd Department of Internal Medicine, Research Unit and Diabetes Center, Medical School, Attikon University Hospital, National and Kapodistrian University of Athens, 12462 Athens, Greece; mankor-th@hotmail.com (E.K.); katerinak90@hotmail.com (A.K.); plioutaloukia@gmail.com (L.P.); gdimitr@med.uoa.gr (G.D.D.)

**Keywords:** MASLD, MASH, NAFLD, nutraceuticals, oxidative stress, insulin resistance, metabolic dysfunction

## Abstract

MASLD (Metabolic Dysfunction-Associated Steatotic Liver Disease) is a growing global concern. Nutraceuticals offer an appealing approach by targeting key mechanisms, such as oxidative stress, inflammation, lipid metabolism, and insulin resistance. This narrative review examines the role of various nutraceuticals in MASLD treatment, including silymarin, vitamin E, omega-3, curcumin, berberine, and coenzyme Q10. Some of them show promising biochemical and metabolic changes, while others produce conflicting results due to relevant studies’ design and endpoints. To bridge the gap between research and reality, we summarize the data, create an interpretation heatmap, and develop a practical supplement guide. Regardless of their potential, nutraceuticals should be viewed as add-ons to lifestyle interventions rather than standalone treatments. Future research should focus on well-designed, long-term studies to prove efficacy, dosing, and combination strategies for personalized MASLD management.

## 1. Introduction

Metabolic Dysfunction-Associated Steatotic Liver Disease (MASLD) and its progressive form, Metabolic Dysfunction-Associated Steatohepatitis (MASH), are two increasingly common liver diseases caused by a combination of genetic, metabolic, and lifestyle factors [1]. Lifestyle modification, mainly through diet and exercise, is the cornerstone for addressing these conditions [2]. However, sticking to lifestyle changes is often difficult, and more strategies that can be combined with dietary and physical activity interventions are needed. Nutraceuticals and dietary supplements with antioxidant, anti-inflammatory, and metabolic benefits have emerged as promising adjuncts to mitigate liver fat, inflammation, and slow the progression of MASLD and MASH [3]. Current research focuses on compounds such as green tea extract, coenzyme Q10, omega-3 fatty acids, and probiotics, each with its own pathway and role in liver health.

This review focuses mainly on studies published in the last ten years, providing an up-to-date synthesis of evidence regarding nutraceuticals in MASLD. Furthermore, we present visual tools, such as tables and a heatmap, to enhance data interpretation and clinical application. A novel, time-structured supplementation guide further bridges research findings with real-world practice, differentiating our work from prior descriptive reviews. We categorize nutraceuticals as plant-based compounds, antioxidants, and metabolic modulators, and examine their mechanisms of action, clinical outcomes, and areas for further research. To provide a well-rounded perspective, this review includes evidence from randomized controlled trials (RCTs), meta-analyses, and observational studies. While RCTs are the gold standard for determining efficacy, observational data also contribute valuable insights, especially in cases where RCTs are limited. For each nutraceutical, we specify the type of evidence supporting its use, and emphasize the need for more RCTs to validate promising observational findings. By collating these findings, we provide clinicians and researchers with an update on the therapeutic potential of these compounds, as well as evidence-based recommendations and future directions for non-pharmacological liver health.

## 2. Methods

In this review, two independent reviewers (E.V. and E.K.) searched the literature in the scientific databases PubMed and Scopus using the following search terms: non-alcoholic fatty liver disease, non-alcoholic steatohepatitis, metabolic dysfunction-associated steatotic liver disease, metabolic dysfunction-associated steatohepatitis, nutraceuticals, polyphenols, antioxidants, metabolic modulators, oxidative stress, insulin resistance, and metabolic dysfunction. The search extended from each electronic database’s inception to 1 January 2025. The studies that were considered eligible for the present review were those that examined the effects of nutraceuticals and dietary supplements in MASLD and MASH. The exclusion criteria comprised studies with a publication date prior to 1 January 2015 as well as studies written in a language other than English. In addition, editorials, case reports, commentaries, pre-prints, non-reviewed studies and conference abstracts were excluded. The current study was thoroughly assessed for potential sources of bias that could affect the validity of the results. Two independent reviewers (E.V. and E.K.) examined the included studies and each study was reviewed by a different person. Any disagreements between reviewers were discussed and resolved by senior reviewers (G.D.D. and V.L.). Due to the study design (narrative review), no specific tools were used for evaluating the methodological quality, including the heterogeneity and risk of bias.

## 3. Plant-Based Nutraceuticals and Polyphenols

### 3.1. Green Tea

#### 3.1.1. Background

Green tea has been used for nearly 5000 years in traditional Chinese medicine to sharpen the mind, support digestion, and promote overall health. Its reputation later spread beyond Asia, and scientific research has since confirmed its potential therapeutic role in managing modern metabolic diseases such as MASLD [4,5].

#### 3.1.2. Mechanism of Action

Green tea’s main active compound is epigallocatechin gallate (EGCG), an antioxidant and anti-inflammatory catechin that has been shown to help manage MASLD. EGCG acts by neutralizing reactive oxygen species (ROS), which are abundant in MASLD and cause liver cell damage and inflammation. It stimulates the activity of key antioxidant enzymes such as glutathione peroxidase and superoxide dismutase, which protect liver cells from oxidative stress [4]. EGCG also modulates lipid metabolism pathways, lowers triglyceride levels, and improves liver enzyme profiles [4].

#### 3.1.3. Clinical Efficacy

In a recent meta-analysis by Mansour-Ghanaei et al. [6], data from four studies showed that green tea supplementation significantly reduced alanine transaminase (ALT) (−12.81 U/L; 95%Confidence Interval (CI): −18.17 to −7.45) and aspartate transaminase (AST) levels (−10.91 U/L; 95% CI: −19.66 to −2.17) in MASLD patients. Additionally, green tea supplementation improved body mass index (BMI) (−2.08 kg/cm^2^; 95% CI: −2.81 to −1.36), triglycerides (TG) (−31.87 mg/dL; 95% CI: −40.62 to −23.12), total cholesterol (TC) (−27.57 mg/dL; 95% CI: −36.17 to −18.98), and low-density lipoprotein (LDL) cholesterol (−14.15 mg/dL; 95% CI: −23.69 to −4.60). Lastly, a systematic review and meta-analysis by Mahmoodi et al. [7], which included 15 randomized controlled trials, found that green tea supplementation significantly reduced liver enzymes in MASLD patients (ALT: Standardized Mean Difference (SMD) = −0.47, 95% CI: −0.70 to −0.25, *p* ≤ 0.001; AST: SMD = −0.80, CI: −1.08 to −0.52, *p* ≤ 0.001).

#### 3.1.4. Lifestyle Integration

Green tea consumption causes relatively few side effects. However, excessive amounts of EGCG can induce mild gastrointestinal symptoms, such as nausea and vomiting, especially when consumed on an empty stomach [4]. For MASLD individuals, drinking 3–5 cups of green tea per day, which contains approximately 200–400 mg of EGCG, is a safe and potentially effective dose [5]. For maximum benefits, consume green tea between meals, as catechins such as EGCG can inhibit iron absorption when taken with food. Patients who are sensitive to caffeine should consider decaffeinated green tea, which contains the same quantity of beneficial catechins [5]. Green tea may serve as a beneficial adjunct within a healthy lifestyle, based on emerging evidence supporting its antioxidant and metabolic effects in MASLD. This integration can improve liver health outcomes and overall metabolic wellness, making green tea a practical, evidence-based addition to MASLD treatment [7].

### 3.2. Caffeine

#### 3.2.1. Background

Coffee, originally cultivated in Ethiopia, has been consumed for centuries across cultures for its stimulating and health-promoting effects. Ancient use to relieve digestive issues and fatigue has evolved into modern evidence supporting its therapeutic potential in chronic liver diseases, including MASLD [8,9].

#### 3.2.2. Mechanism of Action

Coffee’s hepatoprotective effects come from bioactive compounds such as caffeine and chlorogenic acids, which have anti-inflammatory and antioxidant properties. Caffeine inhibits the Toll-like receptor 4 (TLR4)/Mitogen-activated Protein Kinase (MAPK)/Nuclear Factor kappa B (NF-Kb) pathway and reduces Nod-like receptor protein 3 inflammasome activation, which is a major cause of inflammation in MASLD and MASH [10,11]. Coffee also upregulates cytochrome P450 enzymes such as CYP7B1, modulates insulin resistance via hepatocyte nuclear factor-4 alpha signaling, and reduces hepatic oxysterol accumulation, which is a precursor to liver damage [12]. Decaf coffee provides some of these benefits, indicating that caffeine-independent mechanisms exist [8].

#### 3.2.3. Clinical Efficacy

A recent meta-analysis by Hayat et al. [13], which included 11 observational studies, found that coffee consumption was associated with a lower risk of MASLD (pooled risk ratio (RR) = 0.77; 95% CI: 0.60–0.98). In patients already diagnosed with MASLD, coffee consumption was associated with a lower risk of liver fibrosis progression (RR = 0.68; 95% CI: 0.68–0.79). A systematic review and meta-analysis by Kositamongkol et al. [9] found that among 3752 MASLD patients, coffee consumption was associated with a reduction in liver fibrosis (odds ratio (OR) = 0.67; 95% CI: 0.55–0.80; *I*^2^ = 3%).

#### 3.2.4. Lifestyle Integration

Too much coffee consumption may disturb sleep, cause anxiety, and increase irritability. Pregnant and lactating women, as well as individuals with arrhythmias, should avoid coffee. When discussing coffee consumption, it is critical to consider the possible negative effects as well as the lack of consensus on safe dosage [14]. Daily consumption of 2–4 cups of coffee has been shown to significantly reduce liver enzyme levels and fibrosis [14], while coffee with a high chlorogenic acid content will enhance these effects. Coffee consumption with meals can improve absorption and reduce postprandial oxidative stress. Decaf coffee is still beneficial for caffeine-sensitive people, albeit less so. Incorporating coffee into a balanced diet and active lifestyle maximizes its therapeutic benefits, particularly for individuals with metabolic syndrome [8].

### 3.3. Curcumin

#### 3.3.1. Background

Curcumin, the primary bioactive compound in turmeric (Curcuma longa), has been used for centuries in traditional Ayurvedic and Chinese medicine to treat inflammatory conditions and promote liver health [15]. Modern research has validated its historical reputation, supporting its potential as a natural treatment for chronic liver diseases such as MASLD [16].

#### 3.3.2. Mechanism of Action

Curcumin works in MASLD by targeting several pathways. It activates the master regulator of lipid metabolism, AMP-activated protein kinase (AMPK), which promotes fatty acid oxidation while inhibiting de novo lipogenesis [17]. It reduces the expression of pro-inflammatory cytokines such as interleukin-6 (IL-6) and tumor necrosis factor-alpha (TNF-α) by downregulating NF-κB, a major driver of inflammation. Its antioxidant properties, including scavenging of reactive oxygen species and increasing glutathione levels, mitigate oxidative stress, which is the heart of liver steatosis and fibrosis [18].

#### 3.3.3. Clinical Efficacy

In a recent meta-analysis by Ngu et al. [19], curcumin supplementation resulted in a 3.53-fold increase in the resolution of hepatic steatosis (95% CI: 2.01–6.22) when compared to placebo, as measured by ultrasonography. Furthermore, Malik et al. [20] found that curcumin was related with a significant reduction in triglycerides (13.22 mg/dL (*p* = 0.02)), and waist circumference (4.87 cm (*p* = 0.008)). A meta-analysis by Safari et al. [18] found that 12 weeks of phytosomal curcumin supplementation significantly improved liver fibrosis and steatosis scores, particularly when combined with diet interventions. Finally, Lukkunaprasit et al. [17] demonstrated significant reductions in ALT levels (5.61 U/L (95% CI: −9.37 to −1.85)).

#### 3.3.4. Lifestyle Integration

Curcumin is an antioxidant and anti-inflammatory supplement, largely without adverse effects [16]. Effective doses for MASLD range from 250 mg to 3000 mg per day, with advanced formulations such as phytosomal curcumin demonstrating improved bioavailability. Curcumin formulations that are phytosomal or encapsulated have a higher absorption rate and therapeutic effect [18]. An intervention period of 8 to 12 weeks was found to be associated with significant changes in liver enzymes, lipid profiles, and anthropometric indices [15]. To maximize curcumin’s liver benefits, combine it with a Mediterranean diet rich in vegetables, whole grains, and healthy fats. Regular aerobic exercise is an added benefit [17].

### 3.4. MilkThistle (Silymarin)

#### 3.4.1. Background

Silymarin, derived from the seeds of milk thistle (Silybum marianum), has been used since ancient Greek medicine to treat liver disorders. Dioscorides first described its use for liver problems in the first century AD [21]. By the nineteenth century, German scientists had developed standardized milk thistle extracts, and silibinin—its major active component—was identified as responsible for hepatoprotective effects [21]. Today, silymarin is recognized as a safe and well-tolerated botanical treatment for liver diseases, including MASLD.

#### 3.4.2. Mechanism of Action

Silymarin’s hepatoprotective and therapeutic effects are mediated by multiple biochemical and molecular pathways. One of the primary mechanisms is the activation of the nuclear factor erythroid 2-related factor 2 (Nrf2) pathway, which improves antioxidant defenses by increasing the expression of enzymes such as superoxide dismutase (SOD) and catalase. These enzymes play an important role in reducing oxidative stress and stabilizing mitochondrial membranes, thereby preventing lipid peroxidation [22]. Silymarin inhibits pro-inflammatory cytokines such as TNF-α, IL-6, and interleukin-1β by targeting the NF-κB pathway. This reduces hepatic inflammation, which is a major cause of fibrosis progression [23]. It also directly scavenges ROS and neutralizes lipid peroxides, providing additional protection against oxidative stress. It also inhibits the activation of hepatic stellate cells, which play an important role in fibrosis development.

#### 3.4.3. Clinical Efficacy

Recent studies and meta-analyses have shown silymarin’s effectiveness in managing MASLD. A systematic review by Li et al. [23] found that silymarin supplementation significantly decreased ALT (SMD: −12.39; 95% CI: −19.69 to −5.08) and AST (SMD: −10.97; 95% CI: −15.51 to −6.43) levels. Liver histology improvements were also seen with an OR of 3.25 (95% CI: 1.80–5.87) for hepatic steatosis resolution compared to controls. Another meta-analysis by Malik et al. [24] reported TG reduction (Mean difference (MD): −22.60 mg/dL; 95% CI: −23.83 to −21.38) and high-density lipoprotein (HDL)-cholesterol increase (MD: +2.13 mg/dL; 95% CI: +1.60 to +2.66), along with an improvement in transaminase levels. Lastly, in a clinical trial by Jaffar et al. [25], silymarin cookies decreased ALT and AST levels (from 64.39 to 49.38 U/L and from 61.53 to 45.38 U/L respectively). The same study revealed improvements in inflammatory markers, such as C-reactive protein (CRP), which decreased from 6.32 to 3.39 mg/L, and erythrocyte sedimentation rate values (from 38.72 to 23.86 mm/h).

#### 3.4.4. Lifestyle Integration

A recent systematic review of silymarin points to a low number of reported adverse events (<4%, lower than placebo) and no serious adverse events [21]. The optimal dose of silymarin ranges from 420 to 600 mg per day, preferably divided into three doses [21]. Advanced forms like silymarin phytosomes increase bioavailability so the silymarin can be more effectively absorbed and utilized by the liver cells [22]. Silymarin-fortified foods, such as cookies, have been explored as a practical approach to delivering hepatoprotective compounds; however, further studies are needed to confirm their clinical utility [25]. For enhanced efficacy, silymarin can be paired with a Mediterranean diet which complements its antioxidant and anti-inflammatory properties and overall metabolic health [23].

### 3.5. Berberine

#### 3.5.1. Background

Berberine, a natural alkaloid found in Coptis chinensis, has been used for thousands of years in traditional Chinese medicine to treat gastrointestinal disorders, infections, and metabolic imbalances [26]. Modern research supports its role as a promising agent for managing metabolic diseases such as MASLD.

#### 3.5.2. Mechanism of Action

Berberine treats MASLD through AMPK activation, which mitigates lipogenesis and promotes lipid oxidation in hepatocytes, directly addressing liver fat accumulation. Also, berberine alters gut microbiota, increasing beneficial bacteria while decreasing pro-inflammatory bacteria. This enhances intestinal barrier function, lowers endotoxemia, and reduces systemic inflammation [26]. It also modulates glucose and lipid metabolism by suppressing gluconeogenesis, enhancing insulin sensitivity, and resolving important metabolic derangements in MASLD patients [27].

#### 3.5.3. Clinical Efficacy

Recent research has demonstrated berberine’s effectiveness on MASLD-related parameters. A systematic review by Koperska et al. [27] showed that berberine increases insulin sensitivity and reduces liver fat. A recent systematic review and meta-analysis by Nie et al. [28], which included 10 RCTs and 811 participants, found significant decreases in lipid markers such as TG (SMD: −0.59, 95% CI: −0.86 to −0.31), TC (SMD: −0.74, 95% CI: −1.00 to −0.49), and liver transaminases, and improved metabolic profiles, including Homeostasis model assessment-estimated insulin resistance (HOMA-IR) (SMD: −1.56, 95% CI: [−2.54, −0.58], *p* = 0.002) and BMI (SMD: −0.58, 95% CI: [−0.77, −0.38]). A clinical systematic review by Ionita-Radu et al. [29] reported measurable reductions in liver fat along with improved serum lipid and hepatic enzyme profiles. Additionally, a randomized trial by Harrison et al. [30] investigated the effects of berberine ursodeoxycholate, demonstrating efficacy with a 4.8% reduction in liver fat content as measured by magnetic resonance imaging, alongside improved glycemic control, reductions in liver enzymes, and weight loss.

#### 3.5.4. Lifestyle Integration

Berberine has no toxicity in standard doses and is clinically beneficial with minimal side effects. The only reported side effects were mild gastrointestinal reactions that occurred on occasion [27]. Berberine doses of 500–1500 mg/day have been shown to benefit MASLD and related metabolic disorders. These doses are frequently split into 2–3 doses a day to increase bioavailability while keeping stable plasma levels [28]. A more advanced formulation, berberine ursodeoxycholate, was tested at 1000 mg twice a day and demonstrated a significant reduction in liver fat [30]. Dietary changes are strongly advised to enhance berberine’s effects. Berberine may be used in conjunction with the Mediterranean diet, which is high in antioxidants, fiber, and healthy fats, to improve lipid profiles and reduce oxidative stress.

### 3.6. Artichoke

#### 3.6.1. Background

Artichoke (Cynara scolymus) has been used for centuries in Mediterranean traditions for its hepatoprotective and lipid-lowering effects. Its active compounds, cynarin and chlorogenic acid, exhibit antioxidant and hepatoprotective properties. Modern research supports its role as a potential intervention for metabolic diseases such as MASLD [31].

#### 3.6.2. Mechanism of Action

Artichoke exerts its hepatoprotective effects through multiple mechanisms. Its polyphenolic compounds (cynarin, chlorogenic acid, caffeic acid, and luteolin glycosides) function as antioxidants, hindering oxidative stress and lipid peroxidation in hepatocytes. It regulates key enzymes such as 3-hydroxy-3-methylglutaryl coenzyme A reductase and sterol regulatory element-binding proteins to increase cholesterol excretion while decreasing de novo lipogenesis and hepatic lipid accumulation [31]. Luteolin also inhibits the Niemann-Pick C1-Like 1 protein, minimizing intestinal cholesterol absorption and systemic lipid levels. All of these actions not only address hepatic steatosis, but they also reduce the inflammatory burden by lowering cytokine production and ROS generation, the drivers of liver fibrosis in MASLD [32].

#### 3.6.3. Clinical Efficacy

In a recent systematic review and meta-analysis by Moradi et al. [33], artichoke supplementation significantly decreased AST and ALT levels (*p* = 0.003 and <0.001 respectively) compared with placebo in patients with MASLD and among overweight or obese individuals. A systematic review and meta-analysis of five RCTs with 333 patients by Kamel et al. [34] demonstrated significant reductions in ALT and AST levels (*p* < 0.001 for both) in patients receiving artichoke leaf extract (ALE). This study also showed improvements in lipid profiles, with reductions in TC (*p* = 0.004), LDL-cholesterol (*p* < 0.001), and TG (*p* < 0.001). Furthermore, in a recent randomized controlled trial by Panahi et al. [31] with 90 MASLD patients, ALE treatment significantly improved hepatic vein flow (*p* < 0.001), reduced portal vein diameter (*p* < 0.001) and liver size (*p* < 0.001), and decreased serum ALT and AST levels (*p* < 0.001). Additionally, ALE supplementation lowered TC, LDL, non-HDL, and TG levels (*p* = 0.01) compared to placebo. Finally, a recent randomized controlled trial by Majnooni et al. [32] studied the combination of ALE, metformin, and vitamin E over 12 weeks in patients with MASLD. The study showed significant reductions in ALT and AST levels (*p* < 0.05), along with improved ultrasonographic findings demonstrating decreased hepatic fat accumulation.

#### 3.6.4. Lifestyle Integration

ALE is considered a safe supplement and has been given in clinical trials with no side effects [31]. A meta-analysis looked at dosing from 100 mg/day to 2700 mg/day for 4–12 weeks and found that baseline cholesterol levels, rather than the dose itself, were more important in determining lipid changes [33]. This flexibility means ALE can be tailored to each individual’s metabolic profile. Lifestyle changes, including diet, are essential for supporting artichoke. A Mediterranean diet rich in antioxidants and healthy fats is often recommended alongside artichoke to reduce oxidative stress and improve lipid metabolism. Exercise and weight management are also important to get the most out of artichoke [31].

## 4. Antioxidants

### 4.1. Vitamin E

#### 4.1.1. Background

Vitamin E was discovered in 1922 by Katherine J. Bishop and Herbert M. Evans during studies on fetal resorption in rats [35]. It includes eight forms, with alpha-tocopherol being the most biologically active due to its antioxidant properties. As a lipid-soluble antioxidant, vitamin E has been used for decades in human nutrition and research, including in the management of MASLD [35].

#### 4.1.2. Mechanism of Action

Vitamin E benefits MASLD due to its antioxidant properties, which break down oxidative stress pathways that cause hepatic inflammation and steatosis [36]. By scavenging ROS, vitamin E inhibits lipid peroxidation and protects hepatocytes from oxidative damage. It also inhibits hepatic de novo lipogenesis (DNL) by regulating transcription factors such as Sterol Regulatory Element Binding Protein 1 (SREBP-1), a critical regulator of lipid metabolism. Vitamin E also improves the activity of antioxidant enzymes such as SOD, which helps to stabilize cell structures. These mechanisms decrease hepatic triglyceride accumulation and inflammation, slowing the progression of MASLD [36].

#### 4.1.3. Clinical Efficacy

In a recent study by Qi et al. [37] with 6122 participants, dietary and total vitamin E intake was found to be inversely associated with MASLD, with particularly strong effects observed in individuals with hyperlipidemia. A systematic review and meta-analysis by Vogli et al. [38], which included 794 patients from 12 RCTs, demonstrated that supplementation with vitamin E at 400 International units (IU)/day led to significant reductions in ALT levels compared with placebo or no intervention (MD: −9.57 IU/L, 95% CI: −12.20 to −6.95) and meaningful reductions in AST levels (MD: −5.60 IU/L, 95% CI: −11.48 to 0.28). A systematic review by Usman et al. [39] highlighted vitamin E’s clinical power in improving liver biochemistry (ALT and AST levels) and histological features (hepatic steatosis and lobular inflammation) in MASLD patients, although its effects on liver fibrosis were less pronounced. Similarly, a meta-analysis by Karedath et al. [40], involving 569 patients, showed significant reductions in ALT, AST, and BMI in the vitamin E group compared to placebo, while no significant differences in fibrosis scores. In a systematic review and meta-analysis by Abdel-Maboud et al. [41], using data from 1317 patients and 15 RCTs, vitamin E supplementation showed superiority in improving ALT, AST, MASLD activity scores and fibrosis outcomes. Lastly, in a study by Vilar-Gomez et al. [42], 800 IU/day of vitamin E was associated with a significant reduction in overall mortality and hepatic decompensation rates in patients with bridging fibrosis and cirrhosis due to MASH.

#### 4.1.4. Lifestyle Integration

Vitamin E at 400–800 IU/day has been shown to be most effective in MASLD patients; however, long-term use at higher doses (≥400 IU/day) has raised some safety concerns, including a slight increase in the risk of hemorrhagic stroke and prostate cancer in certain population studies [42]. Large RCT meta-analyses have not confirmed an increase in overall mortality with doses up to 800 IU/day, suggesting that careful patient selection and monitoring remain key [37,42]. A recent study discovered that the best outcomes are observed in obese individuals, 15–50 years old, with baseline AST > 50 IU/L who take 400–800 IU of vitamin E per day and have the potential of losing 5–10 kg [41]. Another study reported that eating more vitamin E-rich foods (nuts, seeds, and vegetable oils) improves liver function. Vitamin E supplementation, when paired with lifestyle modifications (exercise and weight control), resulted in substantial improvements in metabolic metrics and expanded hepatoprotective benefits [37]. Vitamin E combined with other therapeutic agents (vitamin C or ursodeoxycholic acid) has additional benefits, particularly in reducing oxidative stress and slowing progress to cirrhosis [41].

### 4.2. Coenzyme Q10

#### 4.2.1. Background

Coenzyme Q10 (CoQ10) is a lipid-soluble mitochondrial coenzyme involved in cellular energy production and oxidative phosphorylation [43]. It protects membranes from oxidative damage and supports mitochondrial function. Known for its antioxidant and anti-inflammatory properties, CoQ10 has been explored as an adjunctive therapy for oxidative stress-related conditions, including MASLD [43].

#### 4.2.2. Mechanism of Action

CoQ10 treats MASLD by targeting key pathways. As an AMPK activator, CoQ10 inhibits hepatic DNL and increases fatty acid oxidation, thus reducing hepatic lipid accumulation. This is achieved by downregulating SREBP-1c, acetyl-CoA carboxylase and fatty acid synthase and upregulating peroxisome proliferator-activated receptor alpha (PPARα) and carnitine palmitoyltransferase-1. This shift reduces hepatic steatosis and improves lipid metabolism [44]. CoQ10 also mitigates oxidative stress, a major driver of MASLD progression by scavenging ROS and restoring antioxidant defenses. It reduces inflammatory cytokines and lipid peroxidation markers like malondialdehyde, thereby reducing hepatic inflammation and protecting against further damage [45].

#### 4.2.3. Clinical Efficacy

Ardekani et al. [46], in a systematic review and meta-analysis of RCTs, found no significant reduction in liver enzymes and lipids among MASLD patients who received CoQ10. However, a subgroup analysis of this study suggested that higher doses of CoQ10 and longer follow-up periods resulted in significant improvements in these metrics. In a meta-analysis of 318 participants with metabolic syndrome, Dludla et al. [45] found that CoQ10 supplementation significantly decreased inflammatory markers (*p* < 0.00001) and non-significantly increased adiponectin levels (*p* = 0.07) compared to placebo. In a recent randomized double-blind, placebo-controlled trial by Vrentzos et al. [43], a six-month course of high-dose CoQ10 supplementation (240 mg/day) in MASLD patients improved endothelial and vascular function and reduced hepatic steatosis, but had no significant effect on liver enzymes or fibrosis. Additionally, a randomized controlled trial by Farsi et al. [47] using a lower dose (100 mg/day) found that CoQ10 supplementation significantly decreased liver enzymes, high-sensitivity CRP, TNF-alpha, and the severity of MASLD compared to the control group (*p* < 0.05 for all comparisons). Finally, a randomized controlled trial by Curcio et al. [48] using an even lower dose (20 mg/day) as part of a multicomponent supplement (silymarin, vitamin C, vitamin E, CoQ10, and selenomethionine) reported significant improvements in liver biochemistry, lipid profiles, and ultrasonographic measurements compared to lifestyle interventions alone, although the specific contribution of CoQ10 within this combination remains uncertain. These findings indicate that the efficacy of CoQ10 may be dose- and time-dependent, and not universally observed across all clinical settings, with lower doses potentially contributing to the variability in outcomes observed across studies.

#### 4.2.4. Lifestyle Integration

In supplemented doses, CoQ10 is nontoxic and clinically beneficial, with only minor side effects [43,47]. Most studies on CoQ10 for MASLD have used daily doses ranging from 100 to 240 mg, with higher doses and longer use leading to larger improvements in liver biochemistry and inflammatory markers [43,46]. Combining CoQ10 with other supplements such as vitamin C, vitamin E, silymarin, and selenomethionine has been shown to improve liver health [48]. Lifestyle changes are still required to maximize benefits. To support liver health, it is recommended to follow a Mediterranean diet rich in antioxidants, polyunsaturated fats, and fiber. Regular physical activity tailored to one’s skills enhances CoQ10 by reducing weight and improving insulin sensitivity [43].

### 4.3. Vitamin D

#### 4.3.1. Background

Vitamin D, originally recognized for its role in calcium-phosphorus homeostasis and bone health, is now implicated in a range of metabolic diseases, including MASLD [49]. Early studies linked vitamin D levels to metabolic conditions, leading to investigations into its therapeutic potential in liver health and MASLD management [49].

#### 4.3.2. Mechanism of Action

Vitamin D reduces insulin resistance, which is an important link to MASLD. It achieves this by binding to the vitamin D receptor (VDR) in the liver. VDR activation lowers free fatty acids, which contribute to lipid accumulation and inflammation. VDR also lowers pro-inflammatory cytokines like IL-6 and TNF-alpha, which are elevated in MASLD [50]. Vitamin D enhances insulin sensitivity by reducing oxidative stress and modulating calcium flux, which is essential for insulin action in adipocytes and muscle cells. Calcium dysregulation due to vitamin D deficiency has been linked to decreased Glucose transporter type 4 activity, which worsens insulin resistance [50].

#### 4.3.3. Clinical Efficacy

A systematic review by Sindhughosa et al. [50] showed that vitamin D supplementation significantly improved insulin resistance, with a reduction in HOMA-IR levels (MD: −1.06; *p* = 0.0006), increased vitamin D levels by 17.45 ng/mL (*p* = 0.0002), and modestly reduced ALT levels (MD: −4.44; *p* = 0.02). However, the authors also reported considerable heterogeneity (*I*^2^ = 67%), and no significant improvements were observed for AST levels, suggesting incomplete correction of hepatic injury markers. A meta-analysis by Chen et al. [51] confirmed an increase in vitamin D levels (*p* < 0.05) following supplementation, but found no significant changes in fasting glucose, insulin levels, or HOMA-IR, highlighting a lack of consistent metabolic benefit. Similarly, a systematic review by Wei et al. [52] concluded that vitamin D supplementation reduced inflammation and oxidative stress but did not result in significant improvements in liver enzymes, insulin resistance, glucose metabolism, or lipid profiles in MASLD patients, and pointed to high inter-study variability (*I*^2^ > 75% for most outcomes). In contrast, Rezaei et al. [49] reported beneficial effects of vitamin D supplementation on several metabolic metrics, including reductions in body weight, BMI, waist circumference, ALT levels, and HOMA-IR, along with an increase in HDL-C levels; however, no significant improvements were found for AST, ALP, or GGT, and substantial heterogeneity was observed.

#### 4.3.4. Lifestyle Integration

Vitamin D supplementation at 2000–5000 IU per day is generally safe and well tolerated, and has been shown to improve MASLD, specifically insulin resistance and ALT levels [52], even more so when combined with lifestyle changes such as moderate exercise and a high unsaturated fat, low refined carb diet [51]. Its anti-inflammatory effects enhance when combined with omega-3 fatty acids [52].

## 5. Metabolic Modulators

### 5.1. Omega-3 Fatty Acids

#### 5.1.1. Background

Omega-3 polyunsaturated fatty acids (PUFAs), particularly eicosapentaenoic acid (EPA) and docosahexaenoic acid (DHA), are recognized for their metabolic and anti-inflammatory properties. Found in marine fish, they help lower triglyceride levels and improve lipid metabolism. Imbalances in omega-6 and omega-3 intake, common in Western diets, have been linked to MASLD pathogenesis, suggesting omega-3 PUFAs as a potential therapeutic target [53,54].

#### 5.1.2. Mechanism of Action

Omega-3 PUFAs treat the effects of MASLD by inhibiting SREBP-1, a transcription factor that stimulates de novo lipogenesis and triglyceride synthesis in hepatocytes. They also increase the expression of PPARα, a fatty acid β-oxidation activator and insulin sensitizer. Furthermore, omega-3 PUFAs reduce oxidative stress and lipid peroxidation, both of which are important in the progression of steatosis into MASH. These effects collectively relieve hepatic fat storage and insulin resistance, key drivers of MASLD progression [55].

#### 5.1.3. Clinical Efficacy

In a recent meta-analysis, Aziz et al. [53] found that omega-3 PUFA supplementation significantly improved ALT (MD: −2.12) and AST (MD: −1.50) levels, and led to meaningful changes in TG, LDL, and TC levels. Similarly, a systematic review and meta-analysis by Lee et al. [54], including 22 RCTs with 1366 individuals, showed that omega-3 PUFAs reduced liver fat content (pooled risk ratio 1.52; 95% CI: 1.09–2.13), and improved TG, TC, HDL, and BMI. In another meta-analysis by Musa-Veloso et al. [56], supplementation with omega-3 PUFAs improved ALT, AST, and TG levels, as well as liver fat content, as assessed by liver imaging. Lastly, in a recent trial, Šmíd et al. [57] concluded that 12 months of 3.6 g/day omega-3 PUFA supplementation led to significant reductions in gamma-glutamyl transferase levels, liver fat content, and beneficial lipid changes.

#### 5.1.4. Lifestyle Integration

The observed adverse events were modest and generally well tolerated by patients, with nausea and bloating being the most commonly mentioned, while no significant adverse events occurred [54]. Daily intake of 2–4 g of omega-3 PUFAs, particularly EPA and DHA, appears to be the most extensively investigated dose range for MASLD management [54]. While omega-3 PUFA supplementation has shown promising results in MASLD, lifestyle changes remain the basis of disease management. Based on clinical evidence, weight loss, adherence to a Mediterranean diet, and regular physical activity all play important roles in improving hepatic steatosis and metabolic health. The Mediterranean diet, rich in fish, olive oil, and nuts, has been related to reduced liver fat and inflammation in MASLD individuals [53].

### 5.2. Probiotics and Prebiotics and Synbiotics

#### 5.2.1. Background

The gut–liver axis plays a critical role in MASLD pathogenesis, with gut dysbiosis contributing to increased intestinal permeability and liver inflammation through bacterial toxins like lipopolysaccharides (LPS) [58]. Probiotics, prebiotics, and synbiotics (PPS) have emerged as therapeutic strategies to restore microbial balance, improve gut barrier integrity, and regulate inflammation in MASLD management [59].

#### 5.2.2. Mechanism of Action

PPS benefit MASLD individuals primarily by targeting gut dysbiosis, intestinal permeability, and hepatic inflammation. Gut microbiota imbalances in MASLD are characterized by an increase in pathogenic bacteria (e.g., *Escherichia* spp.) and a decrease in beneficial microbes (e.g., *Faecalibacteriumprausnitzii*), which contributes to microbial translocation and endotoxemia [58]. Elevated levels of LPS trigger TLR4-mediated inflammation, leading to increased hepatic TNF-α expression and fibrosis progression [58]. Probiotic supplementation has been shown to restore gut microbial balance, improve intestinal barrier integrity by increasing tight junction proteins like Zonula Occludens-1 and occludin, and thus reduce bacterial translocation and systemic inflammation [59]. Prebiotics, which selectively stimulate the growth of beneficial bacteria, contribute to short-chain fatty acid production, which boosts glucose metabolism and lipid homeostasis. Synbiotics, which are a combination of probiotics and prebiotics, work together to improve bile acid metabolism by farnesoid X receptor signaling, which controls fat and glucose levels [59].

#### 5.2.3. Clinical Efficacy

In a recent systematic review and meta-analysis, Li et al. [60] analyzed 29 RCTs with 2110 MASLD patients and showed that PPS supplementation significantly reduced fasting glucose levels (*p* = 0.04), HOMA-IR (*p* < 0.00001), and insulin levels (*p* = 0.002), while lowering TC (*p* < 0.00001) and LDL (*p* < 0.00001). Similarly, a network meta-analysis by Kanchanasurakit et al. [61] studied 26 RCTs with 1389 MASLD patients and found that synbiotics were powerful in reducing AST levels (−12.71 IU/L; 95% CI: −16.95, −8.47), ALT levels (−14.46 IU/L; 95% CI: −21.33, −7.59), waist circumference (−2.26 cm; 95% CI: −2.98, −1.54), and TC (−22.23 mg/dl; 95% CI: −29.55, −14.90). Furthermore, Pan et al. [62] analyzed 34 studies with 12,682 participants and found that PPS therapies improved hepatic fibrosis (SMD = −0.31; 95% CI: −0.53, −0.09), AST (SMD = −0.35; 95% CI: −0.55, −0.15) and ALT levels (SMD = −0.48; 95% CI: −0.71, −0.25), and inflammatory markers like TNF-α (SMD = −0.86; 95% CI: −1.56, −0.56). It is worth noting, however, that the observed improvement in fibrosis was largely driven by a single study that did not utilize elastography; in that study, only the APRI score showed borderline statistical significance. Naghipour et al. [63] conducted an umbrella meta-analysis and concluded that PPS therapies significantly reduce TG (*p* < 0.01), TC (*p* < 0.01), and LDL (*p* < 0.01) levels. Lastly, it should be acknowledged that the PPS studies differed widely in the strains used, dosages, and treatment durations—factors that make the overall results difficult to interpret in clinical practice.

#### 5.2.4. Lifestyle Integration

PPS at a reasonable supplemented dosage cause minimal or no adverse effects, mostly as a result of their osmotic features [59]. Clinical practice suggests that at least 12 weeks of PPS supplementation are required to see results, with some studies extending to 24 weeks for long-term effects. Most trials used probiotic capsules containing strains of *Lactobacillus*, *Bifidobacterium*, or *Streptococcus species*, with total daily doses ranging from 10^9^ to 10^11^ CFU [61]. However, probiotic selection should be made cautiously, as certain formulations may not be suitable for all patients, particularly those with advanced liver disease or immunosuppression. An optimal standardized dose has yet to be identified, emphasizing the need for individualized treatment. From a lifestyle perspective, microbial therapy should be paired with dietary changes. Patients who eat fiber-rich, plant-based diets, such as the Mediterranean diet, may see additional benefits. Furthermore, weight management through calorie control and regular physical activity is recommended, as combined interventions perform better than supplementation alone [61,62,63].

## 6. Discussion

This review explores the potential of various nutraceuticals in MASLD management, focusing on liver protection, reducing inflammation, and regulating metabolism. Silymarin, vitamin E, omega-3 fatty acids, curcumin, berberine, and CoQ10 are among the most extensively researched. Although several nutraceuticals show beneficial effects on liver enzymes, lipid profiles, and non-invasive fibrosis markers, histological improvement of fibrosis remains rare. Currently, only high-dose vitamin E has shown evidence of fibrosis regression confirmed by liver histology in RCTs [41,42], highlighting a critical gap in the literature and the need for robust trials using histological endpoints. Vitamin D, which is well known for its role in metabolism, has yielded conflicting results in clinical trials. This suggests that patient-specific factors, such as baseline vitamin D levels and genetic makeup, may influence its efficacy [64].

MASLD, historically known as non-alcoholic fatty liver disease, is a systemic condition which involves cardiometabolic risk factors such as obesity, type 2 diabetes, insulin resistance, and lipid abnormalities in besides hepatic steatosis [65]. Obesity is the leading cause of MASLD given that the liver is the primary organ for glucose and lipid metabolism. Beyond hepatic manifestations, MASLD has been linked to systemic complications such as chronic kidney disease, osteoporosis, obstructive sleep apnea syndrome, endocrine disorders, cognitive impairment, and cardiovascular disease [66]. The connection between MASLD and atherosclerosis is complex, with links to severe lipotoxicity, inflammation, and hepatic insulin resistance. Despite growing awareness of these links, MASLD has received insufficient attention in the medical community, highlighting the need for a multidisciplinary approach [66].

The primary therapeutic goals in MASLD are to minimize liver damage, reduce chronic inflammation and fibrosis, and address the major cardiometabolic risk factors. According to clinical guidelines, lifestyle changes should be implemented first [67]. That includes quitting smoking, losing weight, shifting diet habits, and exercising regularly. High-intensity interval training has been shown to be especially effective for improving lipid profile, insulin sensitivity, and cardiovascular health [67,68].

A growing body of research highlights that nutraceuticals and lifestyle modifications, such as adherence to a Mediterranean diet and structured exercise regimens, may act synergistically through multiple mechanisms to improve health outcomes [69]. Nutraceuticals rich in bioactive compounds complement the antioxidant and anti-inflammatory effects of the Mediterranean diet [69]. Exercise further enhances endogenous antioxidant defenses and reduces systemic inflammation, providing a dual strategy against oxidative stress and chronic inflammation [70]. Synergistic benefits are also observed in metabolic regulation, lipid profiles, insulin sensitivity, and neuroprotection, particularly through pathways involving brain-derived neurotrophic factor [71]. Moreover, the concurrent use of nutraceuticals and exercise may optimize gastrointestinal absorption and tissue delivery of bioactive compounds [72].

Contraindications for nutraceuticals in MASLD remain poorly defined, as many supplements are self-prescribed and their safety profiles are still emerging. Some supplements, such as green tea extract, have been linked to hepatotoxicity [73]. In patients with advanced MASLD, caution is warranted when initiating nutraceuticals. Monitoring with baseline and serial liver function tests is recommended. Potential interactions between nutraceuticals and pharmacotherapy are another critical concern, especially in the older MASLD population with polypharmacy. Supplements considered “natural” can interact with medications used to treat metabolic disorders, such as statins and antidiabetic agents [74]. Although specific interactions in MASLD require further study, clinicians should systematically inquire about supplement use to proactively identify and manage risks.

Nutraceuticals currently occupy a regulatory gray zone between foods and pharmaceuticals. In the United States and many other countries, dietary supplements are regulated as foods without a requirement for pre-market efficacy or safety approval. Although good manufacturing practice regulations aim to ensure quality, manufacturers largely set their own specifications, and compliance monitoring remains limited. Adverse event reporting is required only for serious events and is often incomplete. Long-term safety data for most nutraceuticals are lacking, underscoring the need for post-marketing surveillance and more rigorous clinical trials before widespread clinical adoption [75,76].

Despite the potential benefits of nutraceutical supplementation in MASLD, current guidelines do not unequivocally recommend their use due to a lack of large-scale, conclusive trials demonstrating significant improvements in MASLD outcomes [77]. Nutraceuticals should therefore be regarded as adjunctive to the cornerstone lifestyle interventions emphasized in guidelines. Nonetheless, emerging evidence suggests that supplementation strategies targeting inflammatory pathways—such as modulation of IL-6 and NF-κB expression—could represent a promising future direction for MASLD management [78].

The evidence for nutraceuticals use in managing MASLD is patchy. This is because many studies have small sample sizes, short follow-up periods, and varying formulations of nutraceuticals [68]. It is important to mention that while several nutraceuticals show promise in observational studies, the absence of RCT data limits the strength of current recommendations. Future studies should prioritize randomized controlled designs to confirm efficacy and safety in MASLD populations and reduce bias inherent in non-randomized data.

**Figure 1:**
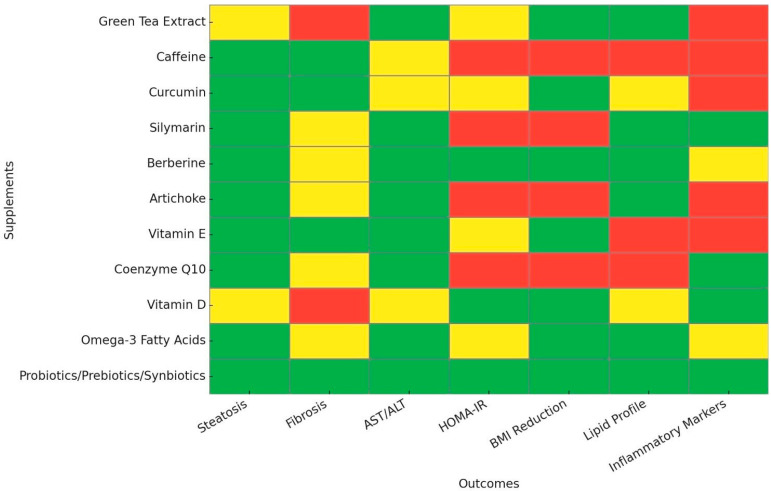
The evidence levels for nutraceutical effectiveness in MASLD management. This heatmap visualizes the strength of evidence supporting different supplements for various clinical outcomes in the management of MASLD. The Y-axis lists the evaluated supplements, while the X-axis presents key clinical outcomes, including steatosis, fibrosis, AST/ALT levels, HOMA-IR, BMI reduction, lipid profile improvement, and modulation of inflammatory markers. Color coding represents the quality and consistency of evidence: Green indicates strong clinical evidence from randomized controlled trials and meta-analyses; yellow indicates limited or inconsistent clinical evidence; and red represents weak, inconsistent, or primarily preclinical evidence. MASLD, Metabolic Dysfunction-Associated Steatotic Liver Disease; AST, Aspartate transaminase; ALT, Alanine transaminase; HOMA-IR, homeostasis model assessment-estimated insulin resistance; BMI, body mass index.

Our summary table of nutraceuticals in MASLD management [Table 1] organizes current research into a simple format, supporting clinical decision-making by detailing key mechanisms of action, expected outcomes, and optimal dosing instructions. The promise for combination nutraceutical therapy, which takes advantage of synergistic interactions among nutraceuticals, is gaining attention [79,80,81,82]. For this purpose, we created a heatmap [Figure 1] that visualizes the evidence levels of various nutraceuticals in MASLD management based on their impact on key metabolic and liver-related outcomes, as analyzed in this review. The color coding was based on the strength and consistency of available clinical evidence used in this review, with priority given to RCTs and meta-analyses. Green was assigned when multiple RCTs and/or meta-analyses consistently supported efficacy. Yellow was assigned when evidence was promising but limited to a small number of RCTs or observational data. Red was assigned when evidence was weak, inconsistent, or based only on preclinical studies or expert opinion without strong clinical data.

The creation of nutraceutical formulations that combine complementary mechanisms, such as antioxidant action, lipid modulation, and gut microbiota regulation, could improve treatment effectiveness. For example, combining omega-3 fatty acids with polyphenols or silymarin with vitamin E may have synergistic benefits for reducing liver inflammation. Personalized supplementation strategies that take into account genetic predispositions and metabolic phenotypes may improve liver outcomes [83]. To put these findings into practice, the ‘A Day in the Life—MASLD Supplementation Guide’ [Table 2] describes an evidence-based plan that matches dietary factors with metabolic rhythms. This table details the optimal timing of meals, drinks, and supplements throughout the day, ensuring that nutrient intake is synchronized with key metabolic processes to maximize liver health.

This review has several limitations that should be acknowledged. First, many of the cited studies enrolled heterogeneous patient populations, including individuals across the MASLD and MASH spectrum and diabetic status. Such variability may influence responses to nutraceutical interventions, complicating direct comparisons across studies. Second, the quality and strength of the available evidence varied, with some nutraceuticals supported mainly by small trials or observational data rather than large-scale randomized controlled trials. Third, dosing regimens, formulations, and treatment durations were inconsistent across studies, further limiting generalizability. Finally, despite efforts to emphasize clinical applicability, this review remains limited by its narrative design and lack of formal meta-analytic methods, which could introduce selection bias and conflicts of interest related to industry-funded research.

## 7. Conclusions

A growing body of evidence indicates that several nutraceuticals, including dietary supplements and herbal extracts, can improve MASLD-related biochemical and histological parameters, suggesting a potential role as complementary therapies. Based on data presented in this review, silymarin, berberine, omega-3 fatty acids, and probiotics/prebiotics/synbiotics emerge as the most promising candidates for MASLD management. However, nutraceuticals cannot replace pharmacotherapy, and their clinical use must follow an evidence-based approach. Long-term, well-designed randomized controlled trials are needed to confirm their efficacy, durability, and safety over time.

Future research should look beyond traditional endpoints, including approaches like nutrigenomics and metabolic phenotyping to better make interventions to individual patient needs. Understanding how genetic variability, microbiome composition, and metabolic signatures influence responses to specific nutraceuticals is critical for developing personalized treatment strategies. Furthermore, clarifying the synergistic effects of combined bioactive compounds, as well as identifying their precise molecular targets and pathways remains a top priority. Although important knowledge gaps remain, emerging clinical data support the use of nutraceuticals within an integrative, precision medicine framework for MASLD management.

## Figures and Tables

**Table 1:** Summary table of nutraceuticals in MASLD management.

Name	Mechanism of Action	Main Outcomes	Dose Recommendation
Green Tea Extract	Anti-inflammatory and antioxidant via EGCG actions	⬇ AST/ALT, ⬇ BMI, ⬇ TC/LDL-C/TG	200–400 mg EGCG daily or 3–5 cups of tea
Caffeine	Anti-inflammatory and antioxidant via TLR4/MAPK/NF-Κb inhibition	⬇ Liver fat, ⬇ Liver fibrosis	2–4 cups of coffee per day
Curcumin	Activates AMPK, reduces inflammation via IL-6/TNF-a inhibition	⬇ Liver fat, ⬇ Liver fibrosis, ⬇ ALT, ⬇ TG, ⬇ BMI	250–3000 mg/day
Silymarin	Antioxidant via Nrf2 activation, anti-inflammatory via IL-6/TNF-a inhibition	⬇ AST/ALT, ⬇ Liver fat, ⬇ TG, ⬆ HDL-C, ⬇ CRP	420–600 mg/day
Berberine	Activates AMPK, modulates gut microbiota, improves insulin sensitivity	⬇ Liver fat, ⬇ AST/ALT, ⬇ TC/TG, ⬇ HOMA-IR, ⬇ BMI	500–1500 mg/day
Artichoke	HMG-CoA & NPC1L1 inhibition, ROS reduction	⬇ Liver fat, ⬇ AST/ALT, ⬇ TC/LDL-C/TG, ⬆ Hepatic vein flow	100–2700 mg/day
Vitamin E	Antioxidant, ROS reduction, SREBP-1 suppression	⬇ AST/ALT, ⬇ Liver fat, ⬇ Liver fibrosis, ⬇ BMI	400–800 IU/day
Coenzyme Q10	AMPK activation, SREBP-1c/ROS downregulation	⬇ Liver fat, ⬇ AST/ALT, ⬇ CRP	100–240 mg/day
Vitamin D	Anti-inflammatory via IL-6/TNF-a inhibition, GLUT-4 upregulation	⬇ ALT, ⬆ HDL-C, ⬇ HOMA-IR, ⬇ CRP, ⬇BMI	2000–5000 IU/day
Omega-3 Fatty Acids	SREBP-1 suppression, PPAR-α activation	⬇ Liver fat, ⬇ AST/ALT, ⬇ TC/LDL-C/TG, ⬆ HDL-C, ⬇ BMI	2–4 g EPA/DHA per day
Probiotics/Prebiotics/Synbiotics	Modulates gut microbiota, reduces systemic inflammation	⬇ AST/ALT, ⬇ TC/LDL-C/TG, ⬇ HOMA-IR, ⬇ Liver fibrosis, ⬇ TNF-a, ⬇ BMI	At least 12 weeks of PPS supplementation

This table summarizes current evidence on the clinical use of nutraceuticals for the management of Metabolic Dysfunction-Associated Steatotic Liver Disease (MASLD). It presents the main mechanisms of action, key clinical outcomes, and dose recommendations for each supplement to assist clinical decision-making. Arrow pointing upward indicates an increase; an arrow pointing downward indicates a decrease. MASLD, Metabolic Dysfunction-Associated Steatotic Liver Disease; EGCG, Epigallocatechin gallate; TLR4, toll-like receptor 4; MAPK, mitogen-activated protein kinase; NF-Κb, Nuclear factor kappa B; AMPK, AMP-activated protein kinase; IL-6, Interleukin-6; TNF-a, tumor necrosis factor-alpha; Nrf2, nuclear factor erythroid 2-related factor; HMG-CoA, 3-hydroxy-3-methylglutaryl coenzyme A; NPC1L1, Niemann–Pick C1-Like 1; ROS, Reactive oxygen species; SREBP-1, Sterol Regulatory Element Binding Protein 1; GLUT-4, Glucose transporter type 4; PPARα, peroxisome proliferator-activated receptor alpha; AST, Aspartate transaminase; ALT, alanine transaminase; BMI, body mass index; TC, total cholesterol; LDL-C, low-density lipoprotein-cholesterol; TG, triglycerides; HDL-C, high-density lipoprotein-cholesterol; CRP, C-reactive protein; HOMA-IR, homeostasis model assessment-estimated insulin resistance; IU, International units; EPA, eicosapentaenoic acid; DHA, docosahexaenoic acid; PPS, probiotics, prebiotics, and synbiotics.

**Table 2:** A Day in the Life—MASLD supplementation guide.

Time	Meal	Drink	Supplements
7:00 AM	Scrambled eggs with avocado & whole-grain toast OR Greek yogurt with walnuts	Cup of Coffee (or decaf if sensitive)	Berberine (1000 mg) and Probiotic capsule (before meal)
10:00 AM	A handful of almonds/sunflower seeds	Green Tea	None
1:00 PM	Grilled salmon or lean chicken with roasted vegetables & quinoa	Water or Herbal Tea	Curcumin (800 mg), Silymarin (140 mg), Vitamin D (2000 IU)
7:30 PM	Baked fish or grilled tofu with steamed greens & whole grains	Water or Chamomile Tea	Artichoke Leaf Extract (300 mg), Omega-3 (2 g EPA/DHA), Vitamin E (400 IU), Coenzyme Q10 (240 mg)

This table presents an evidence-based daily supplementation guide for individuals with MASLD, aligning meals, beverages, and nutraceutical intake with metabolic rhythms to optimize efficacy. The probiotic capsule is suggested to contain strains of *Lactobacillus* and/or *Bifidobacterium* species, with a total dose between 10^9^ and 10^10^ colony-forming units (CFU). MASLD, Metabolic Dysfunction-Associated Steatotic Liver Disease; IU, International Units; EPA, eicosapentaenoic Acid; DHA, docosahexaenoic acid.

## Data Availability

All data related to this research are available within the manuscript.

## Abbreviations

The following abbreviations are used in this manuscript:

MASLD	metabolic dysfunction-associated steatotic liver disease
MASH	metabolic dysfunction-associated steatohepatitis
RCTs	randomized controlled trials
EGCG	epigallocatechin gallate
ROS	reactive oxygen species
ALT	alanine transaminase
CI	Confidence Interval
AST	aspartate transaminase
BMI	Body Mass Index
TG	Triglycerides
TC	Total Cholesterol
LDL	Low-density Lipoprotein
SMD	Standardized Mean Difference
TLR4	toll-like receptor 4
MAPK	mitogen-activated protein kinase
NF-Κb	Nuclear factor kappa B
RR	*risk ratio*
OR	odds ratio
AMPK	AMP-activated protein kinase
IL-6	Interleukin-6
TNF-a	tumor necrosis factor-alpha
SOD	superoxide dismutase
MD	Mean difference
HDL	high-density lipoprotein
CRP	C-reactive protein
HOMA-IR	homeostasis model assessment-estimated insulin resistance
ALE	artichoke leaf extract
DNL	de novo lipogenesis
SREBP-1	Sterol Regulatory Element Binding Protein 1
IU	International units
CoQ10	Coenzyme Q10
PPARα	peroxisome proliferator-activated receptor alpha
VDR	vitamin D receptor
omega-3 PUFAs	Omega-3 polyunsaturated fatty acids
EPA	eicosapentaenoic acid
DHA	docosahexaenoic acid
LPS	lipopolysaccharides
PPS	probiotics, prebiotics, and synbiotics
